# Arabidopsis AMINO ACID PERMEASE1 Contributes to Salt Stress-Induced Proline Uptake from Exogenous Sources

**DOI:** 10.3389/fpls.2017.02182

**Published:** 2017-12-22

**Authors:** Ting Wang, Ying Chen, Min Zhang, Jiugeng Chen, Jie Liu, Huiling Han, Xuejun Hua

**Affiliations:** ^1^Key Laboratory of Plant Resources and Beijing Botanical Garden, Institute of Botany, Chinese Academy of Sciences (CAS), Beijing, China; ^2^College of Life Sciences, University of Chinese Academy of Sciences (UCAS), Beijing, China

**Keywords:** *Arabidopsis thaliana*, amino acid permease, positional cloning, proline accumulation, proline transport, salt stress

## Abstract

Stress-induced proline accumulation in plants is thought to result primarily from enhanced proline biosynthesis and decreased proline degradation. To identify regulatory components involved in proline transport, we screened for *Arabidopsis thaliana* T-DNA mutants with enhanced tolerance to toxic levels of exogenous proline (45 mM). We isolated the *proline resistant 1-1* (*pre1-1*) mutant and map-based cloning identified *PRE1* as *AMINO ACID PERMEASE1* (*AAP1*, At1g58360), which encodes a plasma membrane-localized amino acid permease. *AAP1* expression is induced by salt stress and abscisic acid, but not by proline. In *pre1-1* mutants, a 19-nucleotide deletion in the *AAP1* coding region produced a premature stop codon. When grown on proline-containing medium, *pre1-1* mutants accumulated significantly less proline than did the wild type. Under salt stress, proline uptake decreased significantly in *pre1-1* mutants. By contrast, proline uptake increased significantly in the wild type. These results suggest that AAP1 functions in the increase of proline uptake during salt stress. In addition, proline uptake promotes salt tolerance in Arabidopsis seedlings. We conclude that plants can increase proline accumulation by AtAAP1-mediated proline uptake from exogenous source, which help to improve the salt tolerance of seedlings.

## Introduction

Higher plants accumulate free proline in response to a wide range of environmental stresses, including salinity, drought, low temperatures, heavy metals, pathogens, nutrient deficiency, atmospheric pollution, and UV-B (Saradhi et al., 1995; Hare and Cress, 1997; Siripornadulsil et al., 2002; Ashraf and Foolad, 2007; Verbruggen and Hermans, 2008; Verslues and Sharma, 2010; Wang et al., 2015). Although the precise role of proline remains unclear, proline accumulation can improve plant adaptation to stresses, helping plant cells maintain the integrity of membranes, and affecting the scavenging of reactive oxygen species and storage of nitrogen and energy (Ashraf and Foolad, 2007; Szabados and Savoure, 2010).

In plants, increased proline accumulation following environmental stress primarily results from enhanced *de novo* proline biosynthesis from glutamate and reduced proline degradation (Buhl and Stewart, 1983; Rhodes et al., 1986; Kiyosue et al., 1996; Peng et al., 1996). Increased expression of *PYRROLINE-5-CARBOXYLATE SYNTHASE1* (*P5CS1*) upon environmental stress, more than *P5CS2*, results in higher pyrroline-5-carboxylate synthase activity that facilitates proline biosynthesis from glutamate in *Arabidopsis thaliana* (Szekely et al., 2008). Arabidopsis mutants that have decreased or no expression of *P5CS1* accumulate decreased amounts of proline during osmotic stress (Szekely et al., 2008). By contrast, *P5CS1* overexpression enhances proline accumulation in plants (Kishor et al., 1995; Zhu et al., 1998). The down-regulation of *PROLINE DEHYDROGENASE* (*PDH*) expression reduces proline dehydrogenase activity, thus decreasing proline degradation and promoting proline accumulation upon environmental stress. Arabidopsis mutants that have decreased or no expression of *PDH* accumulate elevated levels of proline during environmental stress (Nanjo et al., 1999; Mani et al., 2002; Ribarits et al., 2007). These observations indicate that P5CS1 and PDH function in accumulation of proline in response to stress. Recently, it has been reported that apart from transcriptional regulation, P5CS1 and PDH1 are likely subjected to post-translational modifications (Bhaskara et al., 2015) under low water potential condition. In addition, cellular redox status has also been found to affect the level of proline accumulation under low water potential stress (Sharma et al., 2013; Shinde et al., 2016) through yet unknown mechanism. Other possible regulation of proline accumulation also include the availability of the procusor molecule, such as glutamate (Skopelitis et al., 2006; Lei et al., 2016), but the precise mechanism is not very well understood.

In addition to the regulation of proline biosynthesis and degradation, proline transport also likely plays a role in proline accumulation, possibly by redistributing proline among organs. Past studies on prokaryotes have suggested a role for proline transport in proline accumulation. For example, upon osmotic stress in *Salmonella typhimurium* and *Escherichia coli*, proline accumulated by uptake from the medium via the ProP and ProU proline transport systems, but the bacteria showed no changes in the rates of proline synthesis or degradation (Csonka, 1989). Similarly, growth of *S. typhimurium* on minimal medium containing high salt (0.8 M NaCl) is markedly enhanced by the addition of either proline or betaine (Booth et al., 1988), suggesting a role for the uptake of these osmolyte compounds in salt tolerance.

Reports in plants also suggest that proline transport could play an important role in proline accumulation. NaCl-induced proline biosynthesis in cell suspensions of a salt-tolerant grass (*Distichlis spicata*) was inhibited in growth medium containing 5.0 mM proline, however, the cells took up exogenous proline (Heyser et al., 1989). In barley (*Hordeum vulgare*), *HvProT* encodes a proline transporter and is rapidly induced in root tissue within 30 min following salt stress, implying that an increase in proline translocation occurs before proline biosynthesis (Ueda et al., 2001). In tomato (*Solanum lycopersicum*), proline accumulation in pollen or induced by salinity is not associated with induced *P5CS* expression, which made the authors to propose that proline transport, among other possibility, might be associated with changes in the intracellular proline levels (Fujita et al., 1998). In addition, LeProT1, a transporter of compatible solutes including proline, may contribute to proline accumulation in pollen (Schwacke et al., 1999). In Arabidopsis, *AtProT2* expression is strongly induced under drought and salt stress (Rentsch et al., 1996). Moreover, studies using *atprot* knockout and *AtProT* overexpression lines concluded that *AtProT1* and *AtProT2* function in proline transport *in planta* because *atprot2* mutants were tolerant to a normally toxic level of proline, and *AtProT1* and *AtProT2* overexpression lines were hypersensitive to exogenous proline (Lehmann et al., 2011). Furthermore, it was found that shoot-applied Pro could greatly enhance root elongation and large amount of proline was built up in the root apex of *pdh1-2* under low water potential, suggesting that proline and/or related metabolites are transported from shoot to root (Sharma et al., 2011).

The amino acid permease (AAP) family in Arabidopsis consists of AAP1-8, which are reported to transport different amino acids (Frommer et al., 1993; Fischer et al., 1995, 2002; Okumoto et al., 2002; Lee et al., 2007; Schmidt et al., 2007; Svennerstam et al., 2008). Several AAP family members, including AAP1, can complement a yeast mutant that lacks eight amino acid transport systems; expression of these AAPs can restore growth on medium containing proline as the sole nitrogen source (Tegeder et al., 2000; Okumoto et al., 2002). In Arabidopsis, AAP1 localizes on the plasma membrane and can import neutral amino acids, including proline, into root tissue (Lee et al., 2007). Previous studies suggested that AAP1 facilitates transport of amino acids only at high concentrations, such as above 50 μM in the soil (Lee et al., 2007; Svennerstam et al., 2011). However, it was recently demonstrated that AAP1 mediates the absorption of glutamic acid and neutral amino acids (including proline) in low concentrations in the soil (Perchlik et al., 2014).

Despite considerable progress in the field, little is known concerning the role of proline uptake or redistribution in proline accumulation and stress adaptation *in planta*. To identify the components regulating proline transport and characterize their function in proline accumulation, we screened for proline-tolerant mutants and isolated the Arabidopsis mutant *proline resistance 1-1* (*pre1-1*), which is tolerant to toxic levels of exogenous proline. Map-based cloning identified *PRE1* as At1g58360, encoding the amino acid transporter AAP1. In this report, we show that AAP1 mediates increased proline uptake in Arabidopsis seedlings under salt stress, which contributes to improved salt tolerance.

## Materials and Methods

### Plant Materials and Growth Conditions

The *A. thaliana* (L.) wild-type, mutant, and transgenic plants used in this study are in the Columbia-0 background. The Arabidopsis seeds were surface-sterilized with 10% sodium hypochlorite for 15 min, rinsed six times in sterile distilled water and sowed in petri dishes (9.0 cm in diameter) on Murashige and Skoog (MS) medium with 2.5% Suc and 0.8% agar (pH 5.8). After being stratified at 4°C for 3 days, seeds were germinated in a growth chamber at 22°C under a 16-h: 8-h, light: dark photoperiod (light intensity 120 μmol photons m^-2^ sec^-1^).

For treatment with various conditions, 12-day-old Arabidopsis seedlings grown on a mesh were transferred into 0.5× MS liquid medium with or without different concentration of proline, NaCl, or abscisic acid for the desired time periods with regular agitation. For measurement of proline content after proline treatment, the seedlings were washed thoroughly and quickly in liquid medium, dried between sheets of filter paper and frozen in liquid nitrogen for subsequent analysis.

For proline uptake experiments, Col and *pre1-1*seedlings are grown on MS medium for 2 weeks and then treated liquid MS medium supplemented with 2.5 mM unlabeled L-proline and 2.5 mM ^15^N-labeled L-proline under salt or control conditions for 10 h. For measurement of proline uptake after treatment, the seedlings were washed thoroughly and dried in 60°C for subsequent isotope ratio mass spectrometry analysis.

### Mutant Isolation and Map-Based Cloning

The Arabidopsis T-DNA insertional mutant pool (Zhang et al., 2005) we used is generated by transforming XVE inducible activation tagging vector (Zuo et al., 2000). For mutant screening, the seeds of the mutant population were germinated and grown horizontally on MS agar plates containing 45 mM proline. The seedlings that grew better were selected at 10 days after germination (DAG). One of the mutants was *pre1-1* (*proline resistant 1-1*), which was allowed to grow to maturity for seed setting.

Because the T-DNA insertion does not co-segregate with the phenotype observed, map-based cloning was performed to clone the mutated gene. To this end, the homozygous *pre1-1* mutant (Columbia background) was crossed with Arabidopsis Landsberg *erecta* to generate F_2_ progenies. Because the difference in root length is faster and easier to follow, F_2_ seeds were grown on plates with 45 mM proline and the seedlings with the longest roots were selected and used as a mapping population. The *pre1-1* mutation was delimited to a 64-kb region within BAC clone X7J on chromosome 1 using a series of PCR-based simple sequence-length polymorphism markers. All eleven candidate genes in the 64-kb region were sequenced from *pre1-1* mutant and this analysis identified a 19-bp deletion in the first exon of At1g58360 in *pre1-1*.

### Plasmid Construction and Transformation of Arabidopsis

For *pre1-1* complementation, a 6518-bp genomic fragment of *AAP1/PRE1* spanning from 2140 bp upstream of the ATG to 687 bp downstream of the TGA was amplified from Arabidopsis genomic DNA using recombinant PCR with two pairs of primers (p-3f & p-3r and p-4f & p-4r) containing *Sma I* and *Xba I* restriction sites (Supplementary Table S1). The amplified fragment was digested with *Sma I* and *Xba I* and cloned into the same sites in the binary vector pCAMBIA1300 (He et al., 2012) to generate *pgPRE1*.

For tissue-specific analysis of *PRE1* promoter activity, a 2584-bp promoter fragment of *AAP1/PRE1* was amplified from Arabidopsis genomic DNA with two primers (p-5f and p-5r) containing *Pst* I and *Bam*H I sites (Supplementary Table S1), digested with *Pst* I and *Bam*H I and cloned into the same sites in a modified pCAMBIA1391 with a *GUS* reporter gene and a *NOS* terminator, resulting in *pPro_AAP1_-GUS*.

For PRE1 subcellular localization, the *PRE1* coding sequence was amplified using primers p-2f and p-2r (Supplementary Table S1), cloned into a modified pJIBIA163-1300 containing a 35S promoter, a *GFP* reporter gene, and a *NOS* terminator, resulting in *p35S-PRE1:GFP*. For measurement of PRE1 protein levels, a 2584-bp *AAP1/PRE1* promoter fragment was amplified from *pAAP1-GUS* with two primers (p-5f and p-5r), and was used to replace the 35S promoter in *p35S-PRE1:GFP*, resulting in *pPro_AAP1_- PRE1:GFP*.

The constructs were introduced into Arabidopsis plants via the Agrobacterium-mediated floral-dipping transformation method (Clough and Bent, 1998). Homozygous T3 plants with a single copy of the transgene were used in subsequent experiments.

### Quantitative PCR Analysis

Total RNA was extracted from ∼12-day-old seedlings as described by Hua et al. (2001), treated with DNase I to remove any remaining genomic DNA, and reverse-transcribed using SuperScript II (Invitrogen) according to the manufacturer’s recommendations. Quantitative real-time PCR was performed using a SYBR Green Real-Time PCR Master Mix (Toyobo, Tokyo, Japan) on a Mx3000P thermocycler with an initial denaturation at 95°C for 2 min followed by 40 cycles of denaturation at 95°C for 15 s, annealing at 57°C for 15 s, and extension at 72°C for 15 s. *ACTIN7* was used as an internal standard to normalize the transcript level of target genes. A melting curve was run after the PCR cycles. Reactions were repeated three times for each sample. The *AAP1*-specific primers used were p-6f & p-6r and the *ACTIN7*-specific primers were *ACTIN7*-F and *ACTIN7*-R (Supplementary Table S1).

### Preparation of Protein Extracts and Immunoblot Analysis

Total protein was extracted from transgenic plants containing the construct *Pro_AAP1_-AAP1CDS-GFP* using cell lysis buffer for western blot and immunoprecipitation (Beyotime P0013). Immunoblot analysis was performed using an anti-GFP antibody (Abmart) and Anti-Mouse IgG (Abmart).

### Proline Content Determination

Samples stored at -80°C were ground in liquid nitrogen and then measured for free proline (Pro) content by a colorimetric assay as described by Bates et al. (1973).

### Proline Uptake Determination

For measurement of proline uptake after proline treatment, the seedlings were washed thoroughly and dried for 48 h at 60°C. The dried seedlings were then ground with mixed grinding apparatus (Retsch MM400) and weighed on a microbalance, packaged in tin capsules and analyzed for ^15^N content by isotope ratio mass spectrometry (Thermo Finnigan MAT DELTAplus XP), using an automated nitrogen/carbon analysis system (Flash EA 1112). Un-labeled glycine (with 0.3674 at% ^15^N) was used as the working standard. Results are presented as atom% (N^15^/*N*_total_) and N%. In according to N^15^/*N*_total_ = N^15^/(N^15^+N^14^) = atom% and m_N_ = N^14∗^14+N^15∗^15 = N% ^∗^ m_sample_, the ^15^N content (μg g^-1^ DW) in sample (m_N15_/m_sample_) was calculated by atom %, N% and dry weight.

### Histochemical GUS Staining and GFP Observation

For histochemical GUS staining, *Pro_AAP1_:GUS* transgenic plants were grown in MS medium for 1 week, or in soil until maturation, then whole seedlings or individual organs were immersed into staining solution with *X*-gluc [2 mM *X*-gluc; 10 mM EDTA; 100 mM NaPO_4_ (pH 7.2); 0.5 mM K_4_Fe(CN)_6_; 0.5 mM K_3_Fe(CN)_6_; 0.1% Trix-100], and incubated at 37°C for 2 to 6 h until blue staining was visible. De-coloration was performed in 75% ethanol for 3–5 h.

For observation of GFP fluorescence, protoplasts were isolated as described (Yoo et al., 2007) from *35S-PRE1:GFP* plants grown in soil for 3–4 weeks and observed under an OLYMPUSFV1000 MPE confocal laser scanning microscope.

## Results

### Isolation and Characterization of the Proline-Tolerant Mutant *pre1-1*

**FIGURE 1:**
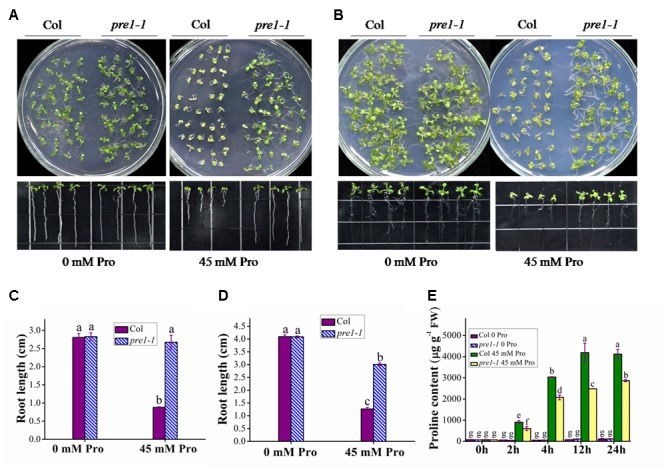
The proline-tolerant phenotype of *pre1-1* mutants. Growth of wild-type (Col) and *pre1-1* seedlings **(A,B)** and average root length **(C,D)** on 0.5× MS medium supplemented with 45 mM L-proline (Pro) following 10 **(A,C)** or 16 **(B,D)** days of growth. **(E)** The proline contents of 12-day-old wild-type and *pre1-1* seedlings treated with 45 mM proline in 0.5× MS liquid medium for various time periods. Experiments were repeated a minimum of three times with similar results. Data are means ± standard error (*n* = 6). Different letters above columns indicate significant difference (*P* < 0.05, Duncan’s multiple range test).

To identify novel components regulating proline transport, we performed a genetic screen for proline-tolerant mutants in a T-DNA insertion mutant population of Arabidopsis. This screen identified the mutant allele *pre1-1* (*proline resistant 1-1*). Whereas there was no significant difference in root length between wild-type (Columbia) and *pre1-1* seedlings grown on MS medium, wild-type root length was reduced to 33 and 42.1% of that of *pre1-1* when grown on MS supplemented with 45 mM L-proline for 10 or 16 days, respectively (**Figures 1A–D**). In addition, the aerial tissue of *pre1-1* mutants was also larger and greener compared to that of wild type. To compare proline uptake in wild type and *pre1-1*, the proline content in 2-week-old seedlings was quantified following treatment with 45 mM L-proline in 0.5× MS liquid medium for various time periods. For each treatment time period, the proline content in *pre1-1* was significantly lower than that of wild type (**Figure 1E**), suggesting that the enhanced proline tolerance of *pre1-1* mutants results from reduced proline uptake.

To characterize the *pre1-1* mutation, the mutant line was back-crossed with wild type (Columbia). The resulting F_1_ (diploid) progeny displayed moderate tolerance to proline (Supplementary Figure S1), suggesting that the *pre1-1* phenotype is caused by a semi-dominant mutation. Examination of the F_2_ progeny revealed segregation of seedlings into longest-root, longer-root, and short-root phenotypic classes, with 70, 156, and 67 seedlines in each respective class [χ^2^(1:2:1) = 1.294 < χ^2^0.05 = 5.99; *P* > 0.05], further demonstrating that the *pre1-1* phenotype is inherited as a semi-dominant trait.

We then determined whether the *pre1-1* mutation is associated with a dominant gain of function or with haplo-insufficiency by crossing homozygous *pre1-1* with tetraploid wild type. The resulting F_1_ (triploid) progeny had a proline-sensitive phenotype similar to that of wild type (Supplementary Figure S1), suggesting that the *pre1-1* phenotype is caused by haplo-insufficiency. Further analysis demonstrated that the proline-tolerant phenotype of *pre1-1* is not linked to a T-DNA insertion. Therefore, individual plants with a proline-tolerant phenotype that did not carry a T-DNA insertion were isolated for map-based cloning of *PRE1*.

### Map-Based Cloning of *PRE1*

**FIGURE 2:**
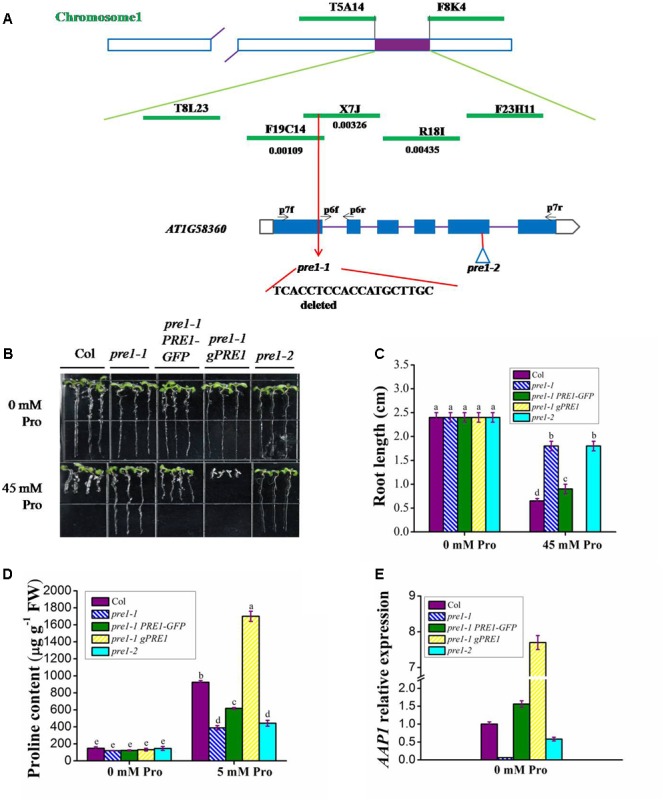
*PRE1* positional cloning and *pre1-1* complementation. **(A)** The *PRE1* locus was mapped to BAC clone X7J on chromosome 1. *PRE1*/*At1g58360*/*AAP1* is composed of 6 exons (black boxes) and 5 introns (lines). BAC names and their regions spanned are indicated. DNA sequencing revealed a 19-bp deletion in the first exon of *PRE1*/*AAP1* in *pre1-1*, resulting in truncation of PRE1/AAP1. The T-DNA insertion site in *pre1-2* is indicated with a triangle. **(B)** Quantification of proline sensitivity and **(C)** average primary root length **(C)** in 9-day-old seedlings of wild type, mutant, and complemented lines treated with 0.5× MS medium supplemented with 45 mM L-proline (Pro). **(D)** The proline content of 12-day-old seedlings of wild type, mutant, and complemented lines treated with 5 mM Pro for 24 h. **(E)** The relative expression of *PRE1/AAP1* in 12-day-old seedlings of wild type, mutant, and complemented lines. Col, wild-type Columbia; *pre1-1 PRE1-GFP*, *pre1-1* transformed *PRE1* cDNA fused with GFP under the control of the *PRE1/AAP1* promoter; *pre1-1 gPRE1*, *pre1-1* transformed with a genomic fragment of *PRE1/AAP1*. Experiments were repeated a minimum of three times with similar results. The values are means ± standard error (*n* = 6). Different letters above columns indicate significant difference (*P* < 0.05, Duncan’s multiple range test).

To clone *PRE1*, we generated an F_2_ population by crossing homozygous *pre1-1* (Columbia) with wild type (Landsberg *erecta*). Since *pre1-1* is a semi-dominant mutant, we obtained the mapping population by sowing F_2_ seeds on MS medium containing 45 mM L-proline and selecting individuals with the longest-root phenotype. Based on classical simple sequence length polymorphism markers, the *PRE1* locus was mapped to the middle of chromosome 1 and was subsequently narrowed down to a 64-kb region within BAC clone X7J and F19C14 (**Figure 2A**). The open reading frames in this region were sequenced in *pre1-1*, which revealed a 19-nucleotide deletion in the first exon of *AT1G58360* (**Figure 2A**), creating a premature stop codon that truncated the encoded protein.

To confirm that *At1g58360* is responsible for the *pre1-1* mutant phenotype, *pre1-1* plants were transformed with either the *AT1G58360* genomic DNA fragment from wild type (*gPRE1*, spanning from 2.5 kb upstream of the start codon to 687 bp downstream of the stop codon) or the *AT1G58360* coding region fused with *GFP* under the control of the *AT1G58360* promoter (*PRE1-GFP*). All complemented lines (*pre1-1 gPRE1* and *pre1-1 PRE1-GFP*) displayed a proline-sensitive phenotype (**Figures 2B,C**) with proline sensitivity directly proportional to the level of *PRE1* transcript, indicating that *AT1G58360* is *PRE1* and functions in proline uptake in a dose-dependent manner (Supplementary Figure S2). We have noticed that *pre1-1 gPRE1* lines are generally more sensitive to proline than are *pre1-1 PRE1-GFP* lines, correlated with the higher level of *PRE1* transcript in the former, despite of same length of the promoter is used in both constructs. The reason for this is currently unknown, but probably related to the presence of intron or 3′UTR in the former and GFP fusion in the later.

*AT1G58360* has been previously annotated as *AAP1*, encoding an amino acid permease family member (Lee et al., 2007), which implies that *pre1-1* is deficient in proline uptake. To confirm the physiological function of PRE1/AAP1, the proline contents of 12-day-old wild-type, *pre1-1*, *pre1-1 gPRE1*, and *pre1-1 PRE1-GFP* seedlings were measured following a 24 h treatment with 5 mM L-proline. As shown in **Figure 2D**, the proline content of *pre1-1 gPRE1* seedlings was significantly higher than that of the other genotypes, which correlated with the significantly higher *PRE1/AAP1* expression observed in *pre1-1 gPRE1* (**Figure 2E**). The proline content of *pre1-1 PRE1-GFP* seedlings was slightly lower than that of wild type, but significantly higher than that of *pre1-1* (**Figure 2D**), which agreed with the proline sensitivity of *pre1-1 PRE1-GFP* (**Figures 2B,C**). The *PRE1/AAP1* transcript level in *pre1-1 PRE1-GFP* plants was comparable to that in wild type (**Figure 2E**).

To further confirm the function of PRE1/AAP1 in proline uptake, a T-DNA insertion mutant of *PRE1/AAP1* (Columbia; GK-324G05-015976) was obtained from the Arabidopsis Biological Resource Center and named *pre1-2*. In this mutant, the T-DNA was inserted at position 1028 (the fifth exon) relative to the putative start codon of *AT1G58360* (**Figure 2A**), and the full-length transcript of *AT1G58360* could not be detected (Supplementary Figure S3). In 9-day-old seedlings, no significant difference was observed in root length between wild type and the *pre1-1* and *pre1-2* mutants grown on MS medium; however, the root length of *pre1-2* was comparable to that of *pre1-1* and approximately fourfold longer than that of wild type, following seedling growth on MS medium supplemented with 45 mM L-proline (**Figures 2B,C**). When treated with 5 mM L-proline for 24 h, the proline content of 12-day-old *pre1-2* seedlings was significantly lower than that of wild type, similar to that observed in *pre1-1* (**Figure 2D**). As in *pre1-1*, *PRE1/AAP1* expression in *pre1-2* was significantly lower than that in wild type (**Figure 2E**). The primers used here are located in the first and second exon (p6f and p6r in **Figure 2A**), so it seems that the partial transcript of *AAP1* in front of the T-DNA insertion can still be detected in *pre1-2*. Since *PRE1* mutation inhibits AAP1-mediated proline uptake, *PRE1* is hereafter referred to as *AAP1*.

### Expression Profile of *AAP1* and Localization of AAP1

**FIGURE 3:**
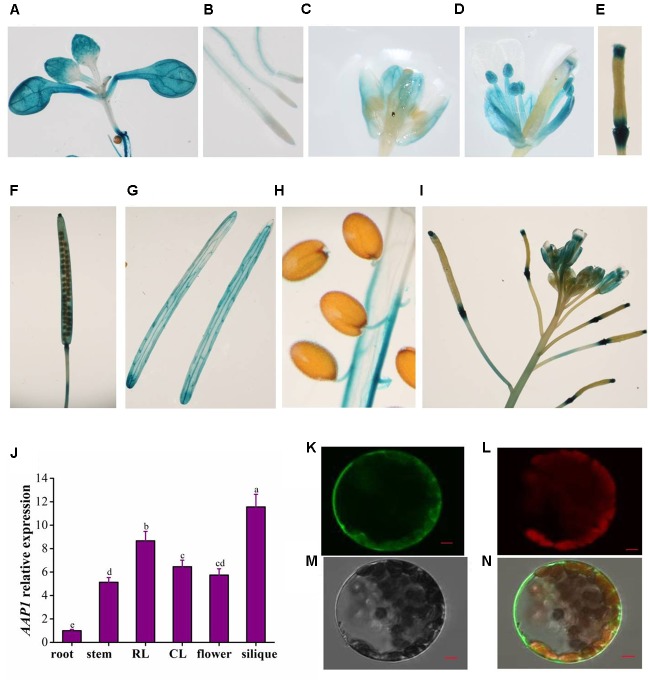
Tissue-specific expression profile of *AAP1* and subcellular localization of AAP1. Histochemical staining of GUS activity directed by the *AAP1* promoter observed in the **(A)** cotyledons and true leaves and **(B)** the root vascular cylinder of 12-day-old seedlings. GUS activity observed in **(C)** flower buds, **(D)** open flowers, **(E)** young siliques, **(F)** developing siliques, **(G)** envelope of mature siliques, **(H)** seed pedicels, and **(I)** silique stalks at the top of the main stem in mature Arabidopsis plants. **(J)** *AAP1* transcript level in different organs of mature wild-type (Col) plants. **(K)** AAP1-GFP fluorescence in protoplasts. **(L)** Chlorophyll auto-fluorescence. **(M)** Bright field. **(N)** Merged image. Bars = 5 μm. Experiments were repeated a minimum of three times with similar results. The values are means ± standard error (*n* = 3). Different letters above columns indicate significant difference (*P* < 0.05, Duncan’s multiple range test).

To characterize the *AAP1* tissue-specific expression pattern, transgenic Arabidopsis plants were generated that carried the β-glucuronidase (*GUS*) gene driven by the *AAP1* promoter (the genomic sequence approximately 2.5 kb upstream of the putative start codon of *AAP1*). Histochemical analysis of GUS activity showed *AAP1* promoter activity in almost all plant organs. In 12-day-old seedlings, *AAP1* promoter activity was observed in cotyledons, true leaves, and roots, with strong activity in the cotyledon and true leaf upper regions, root tips, and the cotyledon and root vascular tissue (**Figures 3A,B**). Relatively less *AAP1* promoter activity was observed in root meristems. In flower buds, *AAP1* promoter activity was primarily concentrated in sepals, and absent in stigmas, filaments, and anthers (**Figure 3C**); however, expression appeared in these tissues in mature flowers (**Figures 3D,I**). During the early stages of silique development, *AAP1* promoter activity was detected at the extremities of siliques (**Figures 3E,I**) and spread throughout the silique tissue as they developed (**Figure 3F**). In mature siliques, strong *AAP1* promoter activity was still visible in the pericarp (**Figure 3G**). No *AAP1* expression was observed in mature seeds, aside from weak expression in the pedicel (**Figure 3H**). Upon silique maturation, *AAP1* promoter activity became stronger in silique stalks (**Figure 3I**). In agreement with the observed GUS activity, the level of *AAP1* transcript was high in siliques and flowers (**Figure 3J**).

To investigate the subcellular localization of AAP1, transgenic Arabidopsis plants were generated that expressed an AAP1-GFP fusion protein under the control of the 35S promoter. Protoplasts isolated from the *AAP1-GFP* plants were observed under a confocal microscope, which revealed GFP localized primarily on the plasma membrane (**Figures 3K–N**), and relatively less GFP signal in the cytoplasm.

**FIGURE 4:**
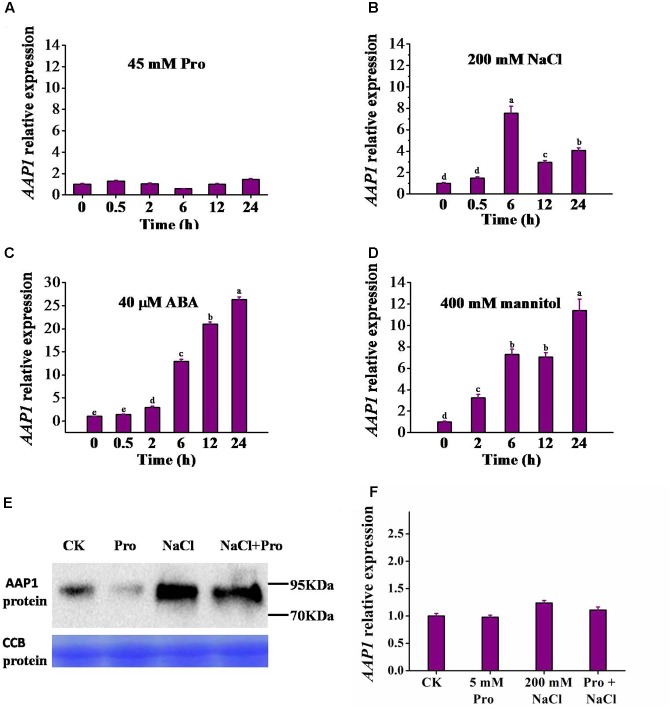
Induction of *AAP1* transcript and AAP1 protein level by different treatments. *AAP1* relative expression level analyzed using real-time RT-PCR in 12-day-old wild-type (Col) seedlings following treatment for different time periods with 0.5× MS liquid medium containing **(A)** 45 mM L-proline (Pro), **(B)** 200 mM NaCl, **(C)** 40 μM abscisic acid (ABA), or **(D)** 400 mM mannitol. **(E)** AAP1-GFP protein level in *35S-PRE1:GFP* seedlings following a 10-h treatment with various compounds. **(F)** *AAP1* relative expression level in *35S-PRE1:GFP* seedlings following a 7 h treatment with 5 mM Pro, 200 mM NaCl, or 5 mM Pro and 200 mM NaCl. *35S-PRE1:GFP*, *pre1-1* transformed *PRE1* cDNA fused with GFP driven by the 35S promoter. Experiments were repeated a minimum of three times with similar results and data are means ± standard error (*n* = 3). Different letters above columns indicate significant difference (*P* < 0.05, Duncan’s multiple range test).

To understand whether *AAP1* expression is responsive to various abiotic stresses that induce proline accumulation, 12-day-old Arabidopsis seedlings were subjected to abiotic stress treatments and *AAP1* expression levels were analyzed using quantitative real-time PCR. The *AAP1* transcript level did not significantly vary over 24 h of treatment with 45 mM L-proline (**Figure 4A**). Similar results were seen with 5 and 10 mM proline (Supplementary Figure S4D). An increase in *AAP1* transcript accumulation was detected upon addition of 50 and 100 mM NaCl (Supplementary Figure S4A). A higher increase was observed after 200 mM NaCl treatment, with an increment peak at 6 h, after which *AAP1* transcript level gradually declined, remaining higher than that in control seedlings at 24 h (**Figure 4B**). *AAP1* expression also rose rapidly following treatment with 40 μM abscisic acid (ABA) and 400 mM mannitol, and at 24 h, reached a level approximately 27- and 11-fold higher than that of control seedlings, respectively (**Figures 4C,D**). 4, 10 μM ABA and 100, 200 mM mannitol can also induce *AAP1* expression, but to lesser extent (Supplementary Figures S4B,C).

Building on the *AAP1* transcriptional up-regulation observed following NaCl treatment, we examined the response of AAP1-GFP fusion protein, under the 35S promoter control, to 10 h treatments with 5 mM L-proline, 200 mM NaCl, or both L-proline and NaCl. For this, we performed western blot experiments using a GFP specific antibody. The 5 mM proline is used here to be consistent with that in salt-induced proline uptake assay later. As shown in **Figure 4E**, the AAP1-GFP level was significantly higher following treatment with 200 mM NaCl or 200 mM NaCl and 5 mM L-proline. However, similar induction of AAP1-GFP was not observed following 5 mM L-proline treatment alone, where it appeared to be reduced compared to that in the control sample. Of note, no significant variation was observed in *AAP1* transcript levels between the different treatments in *35S:AAP1-GFP* seedlings (**Figure 4F**).

### AAP1 Functions in Increased Proline Uptake in Response to Salt Stress

**FIGURE 5:**
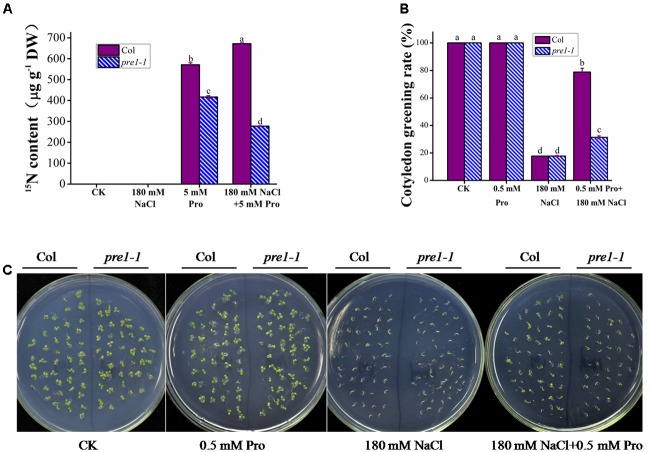
The effect of salt stress treatment on exogenous proline uptake and beneficial effects of increased proline uptake on salt tolerance in Arabidopsis seedlings. **(A)** The proline uptake of wild-type and *pre1-1* 12-day-old seedlings following a 10 h treatment with 0.5× MS liquid medium containing 5 mM L-proline (including 2.5 mM ^15^N labeled L-proline) with or without 180 mM NaCl. **(B,C)** The seeds of wild type (Col) and the *pre1-1* mutant were germinated on 0.5× MS medium containing 180 mM NaCl with or without 0.5 mM L-proline (Pro), grown for 5 days. The survival rate **(B)** (the number of seedlings to compile statistics ≥ 200) of Col and *pre1-1* seedlings and the phenotype **(C)** were measured and photographed, respectively. Col, wild-type Columbia. Experiments were repeated a minimum of three times with similar results. In **(A,B)**, the data are means ± standard error (*n* = 3). Different letters above columns indicate significant difference (*P* < 0.05, Duncan’s multiple range test).

To investigate whether plants can use exogenous proline to increase their internal proline content, we measured the proline uptake in 2-week-old wild-type seedlings grown under salt stress or control conditions following treatment with 5 mM L-proline (including 2.5 mM labeled ^15^N L-proline). Under control conditions, a 10 h proline treatment resulted in a 571 μg g^-1^ DW net increase in seedling proline uptake (**Figure 5A**). However, under salt stress, proline treatment caused a 672 μg g^-1^ DW net increase in seedling proline contents, which was significantly higher than that observed under control conditions (**Figure 5A**).

To investigate whether *AAP1* plays a role in proline uptake during salt stress, *pre1-1* was treated with proline as described above. Similar to the results presented in **Figures 1E**, **5A** shows that the net proline uptake in the *pre1-1* mutant (417 μg g^-1^ DW) was lower than that in wild type under control conditions. Under salt stress, however, the net proline uptake of the *pre1-1* mutant (277 μg g^-1^ DW) significantly decreased and was significantly lower than that of wild type (**Figure 5A**).

To determine whether enhanced proline uptake improves salt stress tolerance in Arabidopsis, *pre1-1* and wild-type seedlings were grown on MS medium containing 180 mM NaCl with or without 0.5 mM proline. Following a 5-day 180 mM NaCl salt stress growing, the rate of cotyledon greening was similar between wild type and *pre1-1* (approximately 18%) on medium without proline, but increased to 80 and 35%, respectively, on 0.5 mM proline-containing medium (**Figures 5B,C**).

## Discussion

### The Proline-Tolerant Mutant *pre1* Affects an Amino Acid Transporter

In this report, we identified and characterized the mutant *pre1-1*, which tolerates levels of exogenous proline that are toxic to wild type. Map-based cloning revealed that *PRE1* encodes AMINO ACID PERMEASE 1 (AAP1) and, in *pre1-1*, a 19-nucleotide deletion in the first exon creates a premature stop codon that likely truncates AAP1. An AAP1-GFP fusion protein localized to the plasma membrane, consistent with previous results (Lee et al., 2007). Previous studies on AAP1 reported that it mediates the uptake of glutamic acid and other neutral amino acids, including proline (Lee et al., 2007). Our results also showed that when grown on medium supplemented with various concentrations of proline, the intracellular proline content of *pre1-1* was significantly lower than that of wild-type Columbia. Furthermore, another mutant line, *pre1-2*, which carried a T-DNA insertion toward the 3′ end of the *PRE1* coding region, displayed a similar proline-tolerant phenotype. These results support the conclusion that AAP1 is a functional proline transporter *in planta*. However, the semi-dominant and haplo-insufficient nature of *pre1-1* indicated that the capacity of AAP1 to transport proline is relatively low. In addition, the complementation lines all showed correlation between proline sensitivity and *AAP1* expression level in a dose-dependent manner. These results are consistent with a previous report that the affinity of AAP1 for proline is low (*K*_m_ = 60 μM) (Lehmann et al., 2010). The *pre1-1* mutant was also more tolerant to other amino acids, such as Met, Val, Phe, Cys, Thr, His, and Ile (Supplementary Figure S5), in agreement with the broad substrate specificity of AAP1 reported previously (Lee et al., 2007).

### AAP1 Is Involved in Mediating the Increase of Proline Uptake during Salt Stress

Notably, our data showed that proline uptake can increase the proline contents in Arabidopsis seedlings under salt stress if proline is available as an exogenous source. Several evidence supported this conclusion. First, in wild type, the seedling proline uptake in the presence of exogenous proline under salt stress was significantly higher than that observed under normal conditions. Second, in the *pre1-1* mutant, the proline uptake under salt stress significantly decreased, suggesting that AAP1 is involved in this process. Lastly, *AAP1* transcript was induced by NaCl treatment and further analyses indicated that the translation and/or the stability of AAP1 was enhanced under salt stress, suggesting that more AAP1 is present in the plasma membrane to facilitate proline transport in response to salt stress. It has been reported that in prokaryotes such as *E. coli*, proline accumulation during osmotic stress primarily involves increased proline uptake from the growth medium, rather than proline biosynthesis (Csonka, 1989). Our results provided evidences that in addition to *de novo* proline biosynthesis and reduced proline degradation, plants can also take up proline from the environment to achieve a higher internal proline content under stress, similar to prokaryotes.

Our results also demonstrate that increased proline uptake improves salt stress tolerance and survival in Arabidopsis seedlings. Improved salt tolerance in wild type compared to that in the *pre1-1* mutant was evident on medium containing proline, whereas, in the absence of proline, similar salt-induced lethality was observed in both wild type and *pre1-1*. We have also noticed that the increase of proline uptake under salt stress is not so dramatic compared to the level of endogenous proline accumulation normally reported, yet still contribute to significant increased salt tolerance of the seedlings. However, we constantly observed this level of increase under our experimental condition. A possible explanation is that the proline taken up during salt stress was not evenly distributed. The local accumulation of proline in specific kinds of cells may exert certain protective effects. Alternatively, proline uptake usually happened faster than its *de novo* biosynthesis, which may be beneficial for early stage growth of the seedling under salt stress.

The protective roles of exogenous proline corroborate previous reports that exogenous application of proline may alleviate the inhibitory effects of salt stress on plant growth (Chen et al., 2011). It has been reported that pretreatment with 0.5 or 5 mM proline significantly improved the growth of Arabidopsis seedlings during a subsequent salt stress treatment (Chen et al., 2011). Exogenous proline supplied at 5–10 mM during stress treatment also increased the salinity tolerance of several plant species and cell cultures (Okuma et al., 2000; Kaya et al., 2007; Teh et al., 2015; Zheng et al., 2015). However, other amino acids have not shown similar protective effects (Okuma et al., 2000). Although a correlation was proposed between the beneficial effect of supplemental proline and both the observed reduction in Na^+^ accumulation (Kaya et al., 2007; Zheng et al., 2015) and the elevation of anti-oxidative capacities (Zheng et al., 2015), no causal relationship has been established.

The toxic effect of exogenous proline under non-stressed conditions has frequently been attributed to a high level of endogenous proline or its catabolic intermediate pyrroline-5-carboxylate (Hellmann et al., 2000; Mani et al., 2002; Miller et al., 2009). A high internal proline level may disrupt the ultrastructure of chloroplasts and mitochondria (Hare et al., 2002). However, the fact that exogenous proline is toxic under normal conditions does not discount its potential protective role under salt stress, since salt stress was found to ameliorate proline toxicity (Hellmann et al., 2000).

AAP1 is a transporter with a known affinity for several neutral amino acids (Lee et al., 2007). Therefore, it is likely that AAP1 mediates uptake of other neutral amino acids, in addition to proline, if they are also present exogenously. However, the salt tolerance of Arabidopsis seedlings was not improved by the presence of other neutral amino acids (Supplementary Figure S6), suggesting that proline has a specific role in alleviating the inhibitory effects of salt stress.

The redistribution of proline among plant organs, which would rely on proline transport, may also be an important salt stress tolerance mechanism. However, we found no difference between wild type and *pre1-1* in proline redistribution among organs during salt-induced proline accumulation (Supplementary Figure S7), suggesting that either AAP1 is not involved in proline redistribution or other transporters may function redundantly in this process. It may be fruitful to characterize other salt-inducible amino acid transporter genes and investigate their possible roles in mediating proline redistribution among organs.

We have demonstrated that treatment with 40 μM ABA significantly increased the level of *AAP1* transcript (**Figure 4C**) and the uptake of proline (Supplementary Figure S8) in wild type. However, in *pre1-1* mutants, ABA treatment did not significantly alter proline uptake (Supplementary Figure S8), suggesting that endogenous ABA might induce proline uptake via *AAP1*. Hormonal regulation of genes encoding amino acid transporters has not been extensively studied, although a study in ginseng showed that expression of the transporter gene *PgLHT1* is induced by ABA, salicylic acid, and methyl jasmonic acid (Zhang et al., 2013). Detailed characterization of mutants deficient in ABA synthesis or ABA responsiveness may produce a greater understanding of the involvement of ABA in *AAP1* expression regulation.

## Conclusion

To explore the contribution of proline transport to proline accumulation under salt stress, we firstly obtained an Arabidopsis mutant *proline resistance 1-1* (*pre1-1*) in a genetic screening for more resistant to toxic level of exogenous proline and identified *PRE1* as At1g58360, encoding the amino acid transporter AAP1, by map-based cloning. Our results indicated that the proline uptake in the presence of exogenous proline under salt stress was significantly higher than that observed under normal conditions in wild type. However, in the *pre1-1* mutant, the proline uptake under salt stress was significantly decreased, which suggested AAP1 is involved in this process. In addition, *AAP1* transcript was induced by NaCl treatment and further analyses indicated that the translation and/or the stability of AAP1 was enhanced under salt stress, suggesting that more AAP1 is present in the plasma membrane to facilitate proline transport in response to salt stress. Analysis of cotyledon greening rate under salinity with or without proline indicated that the growth of wild type seedlings was significantly better than that of *pre1-1* mutant only in the presence of exogenous proline.

Taken together, we conclude that proline transport mediated by PRE1/AtAAP1 is important for proline accumulation under salt stress, which is probably a part of adaptive mechanism for improving salt tolerance.

## Author Contributions

TW, MZ, and XH designed the experiments. TW, YC, MZ, JC, JL, and HH performed the experiments. TW and XH wrote the manuscript. XH revised the manuscript.

## Conflict of Interest Statement

The authors declare that the research was conducted in the absence of any commercial or financial relationships that could be construed as a potential conflict of interest.
