# Supplementary material for: Arabidopsis AMINO ACID PERMEASE1 Contributes to Salt Stress-Induced Proline Uptake from Exogenous Sources
Source: Front Plant Sci. 2017 Dec 22;8:2182. doi: 10.3389/fpls.2017.02182 (PMC5743684; doi:10.3389/fpls.2017.02182)
Supplement: Supplementary file 1 [file Presentation_1.pdf]

Supplementary Material

Arabidopsis AMINO ACID PERMEASE1 contributes to salt stress-induced proline uptake from exogenous sources.

Ting Wang[1, 2], Ying Chen[1, 2†], Min Zhang[1†], Jiugeng Chen[1†], Jie Liu[1, 2], Huiling Han[1, 2], Xuejun Hua [1*]

*** Correspondence:** Xuejun Hua: xjhua@ibcas.ac.cn

1 Supplementary Data

Supplementary Material should be uploaded separately on submission. Please include any supplementary data, figures and/or tables.

Supplementary material is not typeset so please ensure that all information is clearly presented, the appropriate caption is included in the file and not in the manuscript, and that the style conforms to the rest of the article.

2 Supplementary Figures and Tables

For more information on Supplementary Material and for details on the different file types accepted, please see here.

2.1 Supplementary Tables

Supplementary Table S1. Primers used

**Primer name**	**Primer (5′-3′)**
**p-1f (*ACTIN7*-F)**	TGTGCTCGATTCTGGTGATGGT
**p-1r (*ACTIN7*-R)**	TGAGATCCCGACCCGCAAGA
**p-2f**	CACCATGAAGAGTTTCAACACAGAA
**p-2r**	TCACTCATGCATAGTCCGGAAG
**p-3f**	CCCGGGTTACTCACAATTTAATTCT
**p-3r**	TTGAGGCAATTACAATCCACAGTT
**p-4f**	AACTGTGGATTGTAATTGCCTCAA
**p-4r**	TCTAGATAAAAGCCACAACTGTTGC
**p-5f**	CTGCAGTAATGTCTATCGACGTCTT
**p-5r**	GGATCCAGTGATGAGAGAAAGAGAA
**p-6f**	GTCACCGGAAAACGGAATTAC
**p-6r**	GTAACCAACAGTGACCCCAATC
**p-7f**	ATGAAGAGTTTCAACACAGAAGGAC
**p-7r**	TCACTCATGCATAGTCCGGAAG

2.2 Supplementary Figures

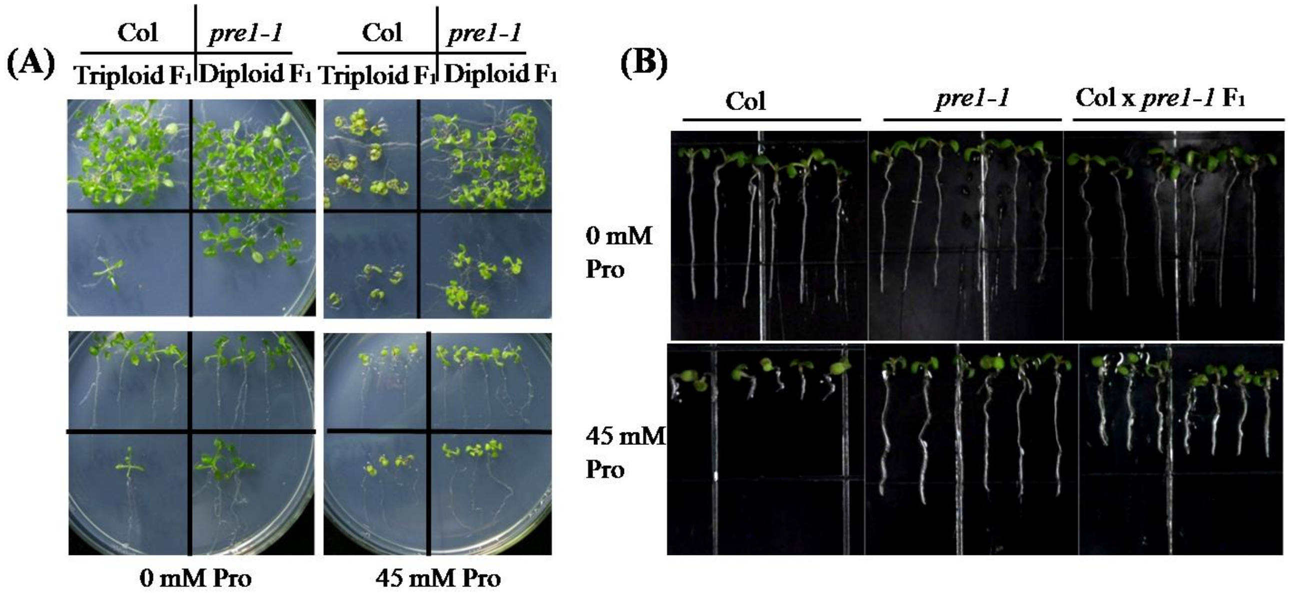

Supplementary Figure S1. The semi-dominance and haplo-insufficiency of the *pre1-1* mutation.

(A) The sensitivity of 12-day-old seedlings of wild-type (Col), *pre1-1*, triploid F_1 (Col tetraploid x *pre1-1* diploid), and diploid F_1 (Col diploid x *pre1-1* diploid) to 45 mM L-proline (Pro). (B) The semi-dominance of the *pre1-1* mutation. Experiments were repeated a minimum of three times with similar results.

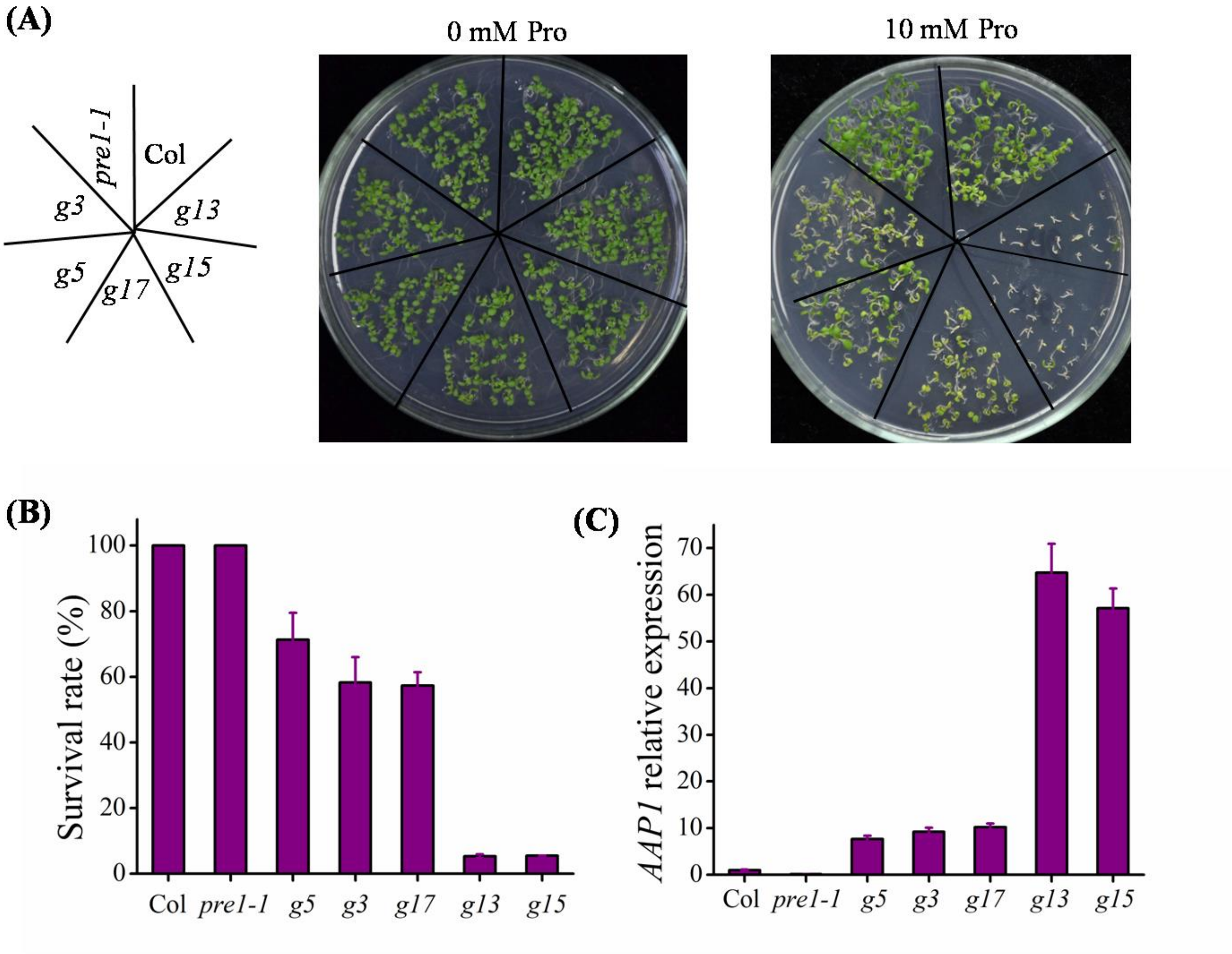

Supplementary Figure S2. Relationship between proline sensitivity and the level of *AAP1* transcript in *pre1-1 gPRE1* complementation lines.

The proline sensitivity (A) and survival rates (B) of 10-day-old seedlings of different complementation lines on 10 mM L-proline (Pro)-containing medium. (C) The *AAP1* transcript level in different complementation lines. Col, wild-type Columbia; *g3*/*g5*/*g17*/*g15*/*g13*: abbreviations for various *pre1-1 gPRE1* complementation lines. Experiments were repeated a minimum of three times with similar results. Data are means ± standard error (n = 3).

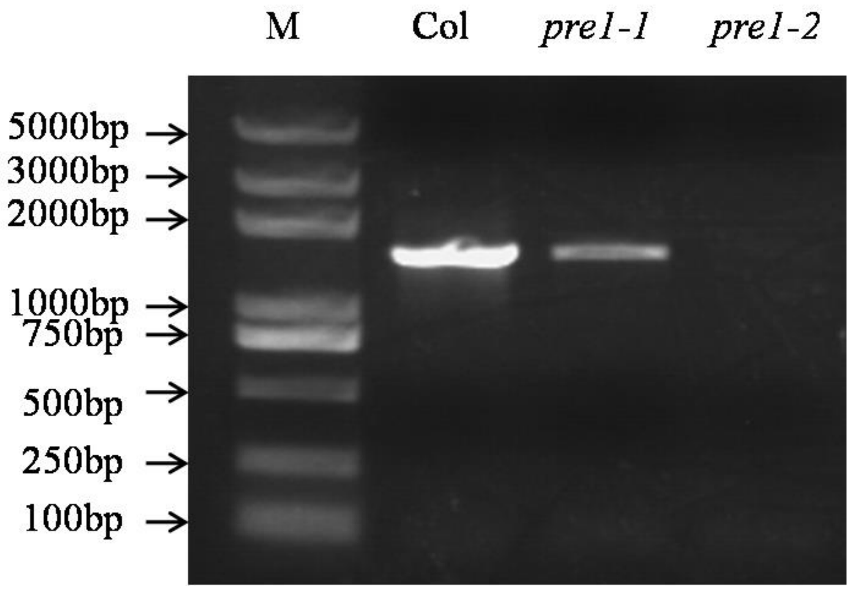

Supplementary Figure S3. Characterization of *AAP1* expression in wild type (Col), *pre1-1*, and *pre1-2* mutant using RT-PCR.

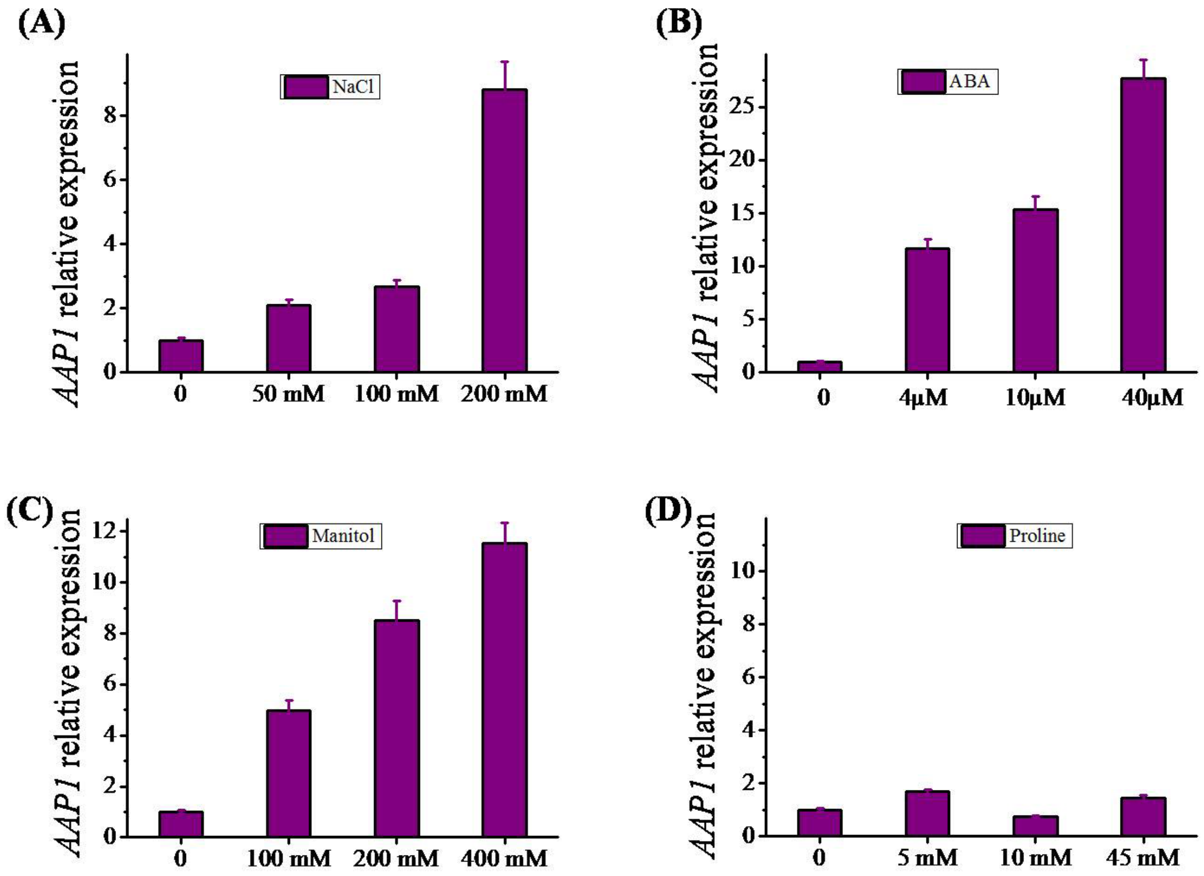

Supplementary Figure S4. Induction of *AAP1* transcript by different treatments.

AAP1 transcript levels were analyzed using quantitative RT-PCR in 12-day-old wild-type seedlings following treatment with 0.5x liquid MS medium containing (A) different concentrations of NaCl for 6 h, (B) different concentrations of abscisic acid (ABA) for 24 h, (C) different concentrations of 400 mM mannitol for 24 h, or (D) different concentrations of L-proline for 24 h. Experiments were repeated a minimum of three times with similar results and data are means ± standard error (n = 3).

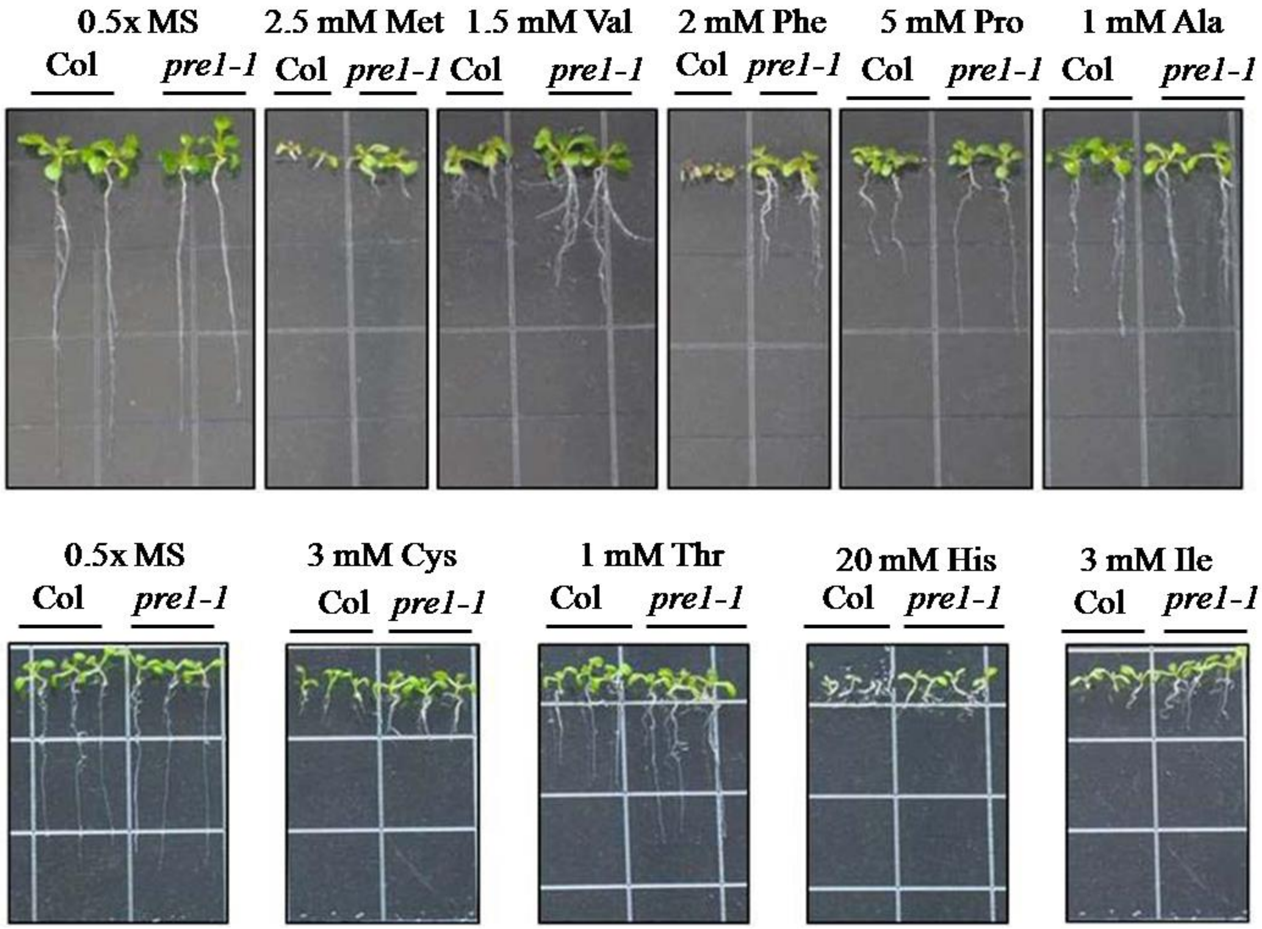

Supplementary Figure S5. Tolerance of *pre1-1* mutants to other amino acids.

14-day-old wild-type (Col) and *pre1-1* seedlings were grown on 0.5x MS medium supplemented with 2.5 mM methionine (Met), 1.5 mM valine (Val), 2 mM phenylalanine (Phe), 5 mM proline (Pro), 1 mM alanine (Ala), 3 mM cysteine (Cys), 1 mM threonine (Thr), 20 mM histidine (His), or 3 mM isoleucine (Ile). Experiments were repeated a minimum of three times with similar results.

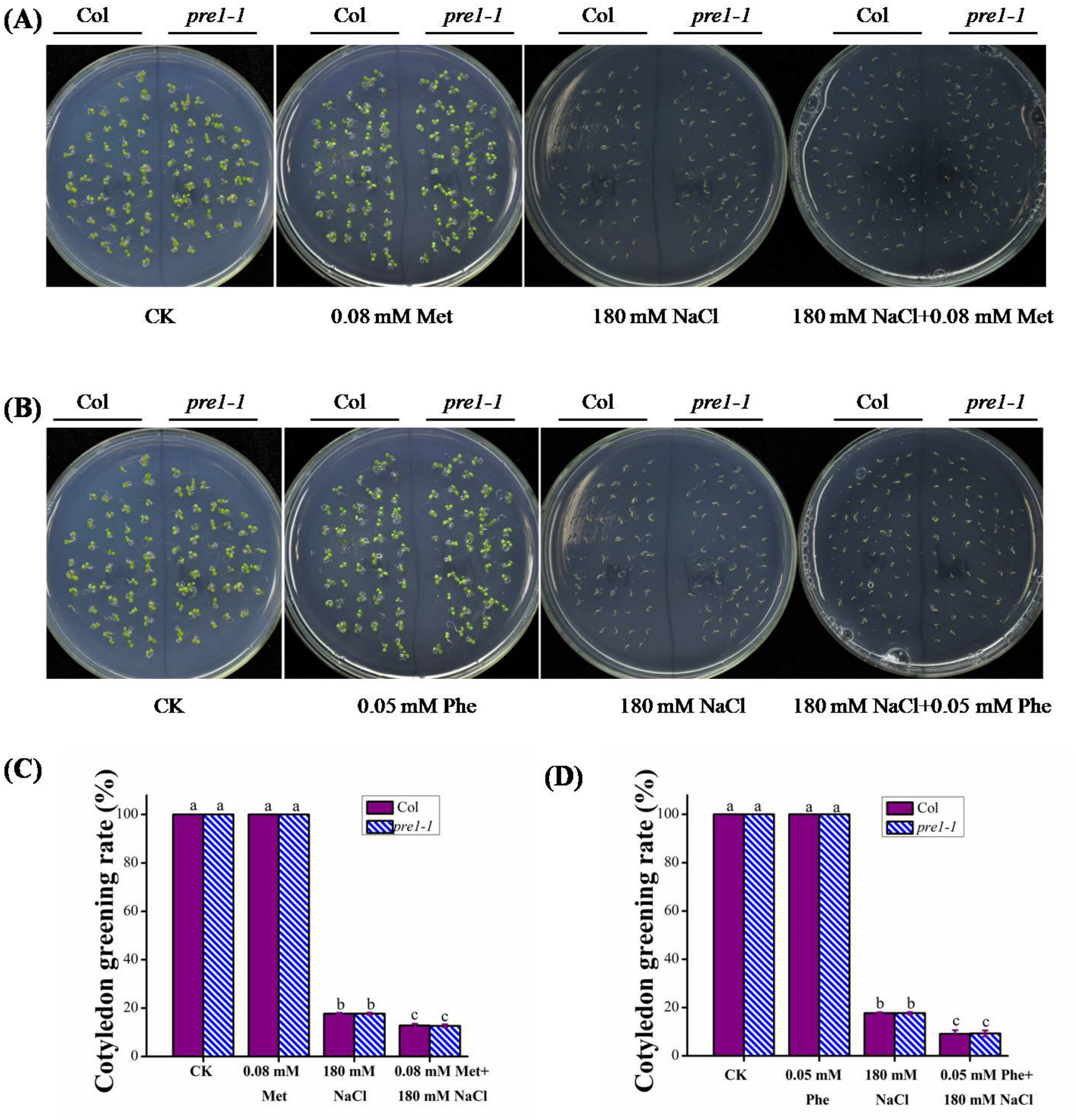

Supplementary Figure S6. The effect of methionine and phenylalanine on salt sensitivity of Arabidopsis seedlings.

The phenotype (A–B) and cotyledon greening rate (C–D) of 5-day-old wild-type (Col) and *pre1-1* seedlings grown on 0.5x MS medium containing 180 mM NaCl with or without methionine (Met) or phenylalanine (Phe). Experiments were repeated at least three times with similar results. The values

are the mean ± standard error (n = 3). Different letters above the columns indicated the significance at $P < 0.05$ according to Duncan's multiple range test.

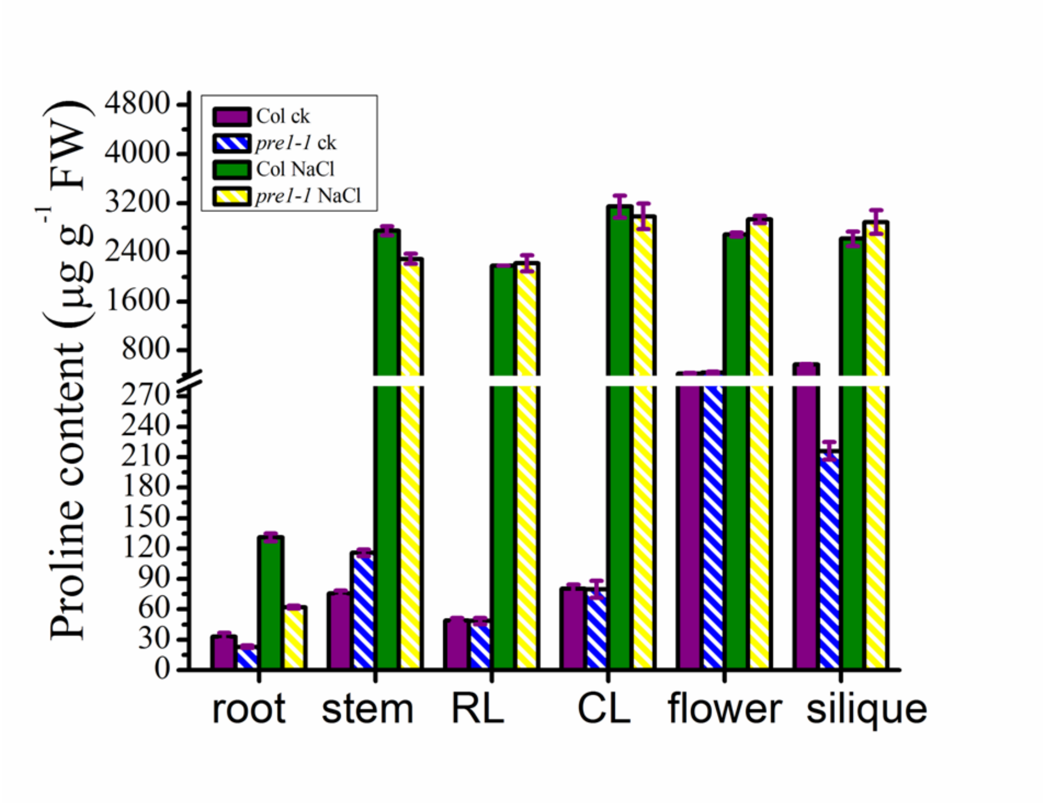

Supplementary Figure S7. The proline contents in different organs of wild-type Columbia (Col) and *pre1-1*.

The 6-week-old hydroponically grown plants were treated with 200 mM NaCl for 24 h and the proline contents in different organs were measured. RL: rosette leaves; CL: cauline leaves. Experiments were repeated a minimum of three times with similar results. Data are means ± standard error (n = 3).

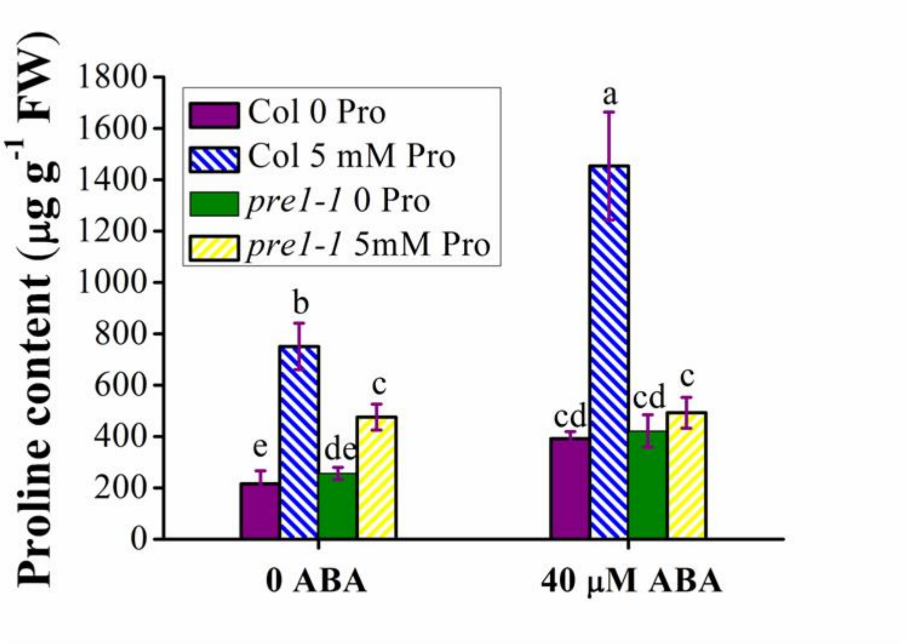

Supplementary Figure S8. The effect of abscisic acid treatment on proline accumulation in different Arabidopsis lines.

The proline contents of wild-type and *pre1-1* 12-day-old seedlings following a 24-h treatment with 0.5x MS liquid medium containing 5 mM Pro with or without 40 μM abscisic acid (ABA). Col, wild-type Columbia. Experiments were repeated a minimum of three times with similar results. Data are means ± standard error (n = 3). Different letters above columns indicate significant difference (P < 0.05, Duncan's multiple range test).
